# Effect of creep feeding pelleted starter diet, liquid milk replacer and a liquid mixture of starter diet and milk replacer to suckling pigs on their growth and medication usage

**DOI:** 10.1093/tas/txae041

**Published:** 2024-03-16

**Authors:** Elisa A Arnaud, Gillian E Gardiner, Matthieu Chombart, John V O’ Doherty, Torres Sweeney, Peadar G Lawlor

**Affiliations:** Teagasc, Pig Development Department, Animal and Grassland Research and Innovation Centre, County Cork, IrelandP61C996; Eco-Innovation Research Centre, Department of Science, South East Technological University, Waterford, IrelandX91K0EK; Eco-Innovation Research Centre, Department of Science, South East Technological University, Waterford, IrelandX91K0EK; Teagasc, Pig Development Department, Animal and Grassland Research and Innovation Centre, County Cork, IrelandP61C996; School of Agriculture and Food Science, University College Dublin, Dublin, IrelandD04V1W8; School of Veterinary Medicine, University College Dublin, Dublin, IrelandD04V1W8; Teagasc, Pig Development Department, Animal and Grassland Research and Innovation Centre, County Cork, IrelandP61C996

**Keywords:** antibiotic, anti-inflammatory, feed intake, large litters, wean, weight

## Abstract

The aim of this study was to assess the effect of creep-feeding solid starter diet, liquid milk replacer, and a liquid mixture of starter diet and milk replacer to suckling pigs on their growth and medication usage up to target slaughter weight (approximately 120 kg). Ninety-one sows and their litters were randomly assigned to one of four post-farrowing treatments at day 107 of gestation; (1) no creep feed provided to weaning at day 28 of age (CONTROL; *n* = 20), (2) dry pelleted starter diet provided as creep feed from day 10 of age to weaning (DPS; *n* = 25), (3) liquid milk replacer provided as creep feed from day 3 of age to weaning (LMR; *n* = 23), and (4) liquid milk replacer provided from days 3 to 6 of age followed by a mixture of liquid milk replacer with an increasing proportion of liquid starter diet to weaning provided as creep feed (LMR + S; *n* = 23). Pig weight and dry matter disappearance (DMd) were recorded during lactation and postweaning until pigs reached target slaughter weight (approximately 120 kg). At target slaughter weight, carcass weight and quality were recorded. Medication (antibiotic and anti-inflammatory) usage per pig on a litter basis, and number of injections and clinical cases of disease per litter were recorded from birth to slaughter. At day 5 postweaning, a subset of pigs (*n* = 40) were sacrificed and intestinal samples were collected for histological analysis. Piglets supplemented with DPS had higher DMd of creep feed than those supplemented with LMR or LMR + S (*P* < 0.001). Providing LMR + S to suckling piglets reduced the coefficient of variation (CV) for within-litter piglet weaning weight (*P* < 0.01) compared to DPS and LMR, but the CV of LMR + S was similar to that of CONTROL. Providing DPS or LMR to suckling piglets increased piglet weaning weight compared to CONTROL (*P* < 0.001) but pig weight was not significantly different from CONTROL at time points thereafter. Gain to feed ratio from weaning to day 6 postweaning was less for LMR pigs compared to all other treatments (*P* < 0.001). Providing DPS or LMR + S to suckling piglets tended to increase postweaning ileal villus height (*P* = 0.07). Diarrhea incidence, as well as the number of clinical cases of disease and injections per litter and volume of antibiotic and anti-inflammatory administered per pig pre- and postweaning, were not affected by treatment (*P* > 0.05). In conclusion, supplementing suckling pigs with liquid milk replacer or dry pelleted starter diet improved growth at weaning, but the benefit did not persist to slaughter.

## Teaser

Providing solid starter diet or liquid milk replacer to suckling piglets increased weaning weight but the benefit did not persist after weaning. Providing a liquid mixture of milk replacer and starter diet to suckling piglets did not benefit pig growth pre- or postweaning.

## Introduction

Weaning is an extremely challenging event for piglets. Pigs are often mixed and moved to an unfamiliar environment. Diet abruptly changes from sows’ milk, a highly digestible diet in liquid form, to a dry solid diet mainly of vegetable origin. This results in delayed and reduced feed intake and growth, and sometimes diarrhea in piglets during the early postweaning period (Hampson and Smith, 1986; Wolter and Ellis, 2001; Collins et al., 2017). Litter size in sows has increased dramatically, particularly in the past decade (+2.5 piglets born alive between 2011 and 2022; Teagasc [2022]). Although demand for milk by the litter increases with increasing litter size, sows have a finite ability to produce milk and therefore, the mean volume of milk available for individual pigs is decreased (King, 2000). This negatively affects piglet preweaning weight gain and weaning weight, as lighter pigs at weaning have reduced lifetime growth and higher mortality rates (Collins et al., 2017). Providing supplementary feed (i.e., creep feed) to suckling piglets is a good strategy to increase preweaning growth and weaning weight. Furthermore, it can familiarize suckling pigs with feed prior to weaning and thereby reduce latency to first feed after weaning and increase postweaning feed intake and growth (Pluske et al., 2007; Muns and Magowan, 2018). However, creep feed consumption varies greatly within and between litters. This is one reason that the response to creep feeding has been found to vary greatly, being particularly influenced by the proportion of ‘eaters’ and ‘non-eaters’ of creep feed within the litter (Bruininx et al., 2002; Sulabo et al., 2010b). Providing supplementary milk to suckling piglets can increase weaning weight and has the potential to increase the proportion of ‘eaters’ within litters (Wolter et al., 2002; Van Oostrum et al., 2016). However, supplemental milk does little to expose piglets to the plant-based ingredients that they will encounter in their postweaning diet. A solution to this is to supplement suckling piglets with a postweaning diet in liquid or gruel form. This has been found to increase piglet feed consumption compared to feeding the same diet in dry form (Martins et al., 2020; Byrgesen et al., 2021).

Increasing creep feed consumption prior to weaning can increase weaning weight (Wolter et al., 2002; Lee and Kim, 2018) and improve intestinal maturity in pigs at weaning (Cabrera et al., 2013; Amdi et al., 2021). Therefore, the practice might be expected to result in more robust pigs at weaning, and as a consequence reduce their need for antimicrobial usage (AMU). However, information is lacking on the effect of creep-feeding strategies on pig intestinal integrity, clinical cases of disease, and medication usage (e.g., AMU). The objective of this study was to determine the effect of providing a supplementary dry pelleted starter diet, liquid milk replacer, and a liquid mixture of milk replacer and starter diet as creep feed to suckling pigs on piglet growth and AMU. Additionally, sow body weight (BW) and back fat (BF) thickness were monitored in order to determine if these strategies could reduce the loss of sow body reserves during lactation. The residual effects of these preweaning dietary treatments on postweaning growth, health, and medicinal usage to target slaughter weight were also determined.

## Materials and Methods

### Ethical Approval

This study was performed between August 2021 and September 2022, at the Teagasc Pig Development Department, Moorepark, Fermoy, Co. Cork, Ireland. Ethical approval was granted by the Teagasc Animal Ethics Committee (approval no. TAEC2020-273) and South East Technological University Ethics Committee (approval no. WIT2021REC011). The project was authorized by the Irish Health Products Regulatory Authority (project authorization no. AE19132/P129). The experiment was conducted in accordance with the legislation for commercial pig production set out in the European communities (welfare of farmed animals) regulations 2010 and in Irish legislation (SI no. 311/2010).

### Experimental Design and Animal Management

Ninety-one sows (Large White × Landrace, PIC®, Hermitage Genetics, Sion Road, Co. Kilkenny, Ireland) were used in this study, which was conducted over four batches (with a batch being a group of sows inseminated on the same week). The first batch included nineteen sows, the second batch included 26 sows, the third batch included 22 sows and the fourth batch included 24 sows. Sows were artificially inseminated at onset of standing estrus and again 24 h later using pooled semen (Topigs Norsvin Tempo, Premier Pig Genetics Limited, Ireland). Gestating sows were housed in dynamic groups of approximately 120 animals. Sows were introduced to the dynamic group between 3 and 6 d after service and fed from electronic sow feeders (Schauer Feeding System [Competent 6], Prambachkirchen, Austria). On day 107 of gestation, sows were blocked within farrowing batch into 25 blocks on the basis of parity group (mean ± SD; 2.4 ± 0.99), number of pigs weaned/sow in the previous cycle (13.4 ± 1.86 for multiparous sows) and BW (270.5 ± 32.61 kg). Sow parity group distribution was as follows: group one, parity 0 (19%); group two, parity 1 to 2 (38%); group three, parity 3 to 5 (25%); and group four, parity 6 to 8 (18%).

Within block, suckling litters were randomly assigned to the following preweaning dietary treatments: (1) no creep feed provided to weaning (CONTROL; *n* = 20), (2) dry pelleted starter diet provided as creep feed from day 10 of age to weaning (DPS; *n* = 25), (3) liquid milk replacer provided as creep feed from day 3 of age to weaning (LMR; *n* = 23), and (4) creep feed provided as liquid milk replacer from days 3 to 6 of age followed by a mixture of liquid milk replacer and liquid starter diet to weaning (LMR + S; *n* = 23). Day 10 was selected as the creep feeding start date for DPS to conform to farm creep feeding practices where creep feeding generally commences after day 10 post-farrowing. However, automated liquid feed delivery systems allow earlier commencement of liquid feeding and we selected to start liquid creep feeding on day 3 after farrowing when piglets had consumed sufficient quantities of colostrum.

Prior to housing of the sows in farrowing accommodation, each farrowing room was cleaned, disinfected, and allowed sufficient time to dry according to standard practices in the facility. At approximately day 110 of gestation, sows were moved into standard farrowing crates in pens (2.5 × 1.8 m) with cast-iron slats under the sow and plastic slats with a water-heated floor pad for the piglets (Big Dutchman, Vechta, Germany). Farrowing room temperature was maintained at 24 ± 3.0 °C at the time of farrowing and gradually reduced to 21 °C by day 7 of lactation. The temperature of the heat pads was 38 to 40 °C for the first 2 d after farrowing and was reduced by approximately 1 °C each day to 30 °C at 10 d after farrowing and it was maintained at this temperature until weaning. Artificial lighting was provided daily from 0800 to 1630 hours. The average number of piglets born alive was 15.9 ± 4.44 piglets. Where possible, litter size was standardized between 24 and 48 h after parturition, with cross-fostering being conducted so that there was an average litter size of 14.6 ± 0.95 piglets per sow at 48 h postpartum. The final number of piglets remaining on each sow at 48 h postpartum was affected by the rearing capacity of each sow (i.e., the number of available functional teats) and the availability of foster sows to take surplus piglets. Piglets’ teeth were clipped within 24 h of birth. On day 5 postpartum, tails were docked and all piglets were injected with 1 mL of iron (Gleptosil, Ceva Santé Animale, Libourne, France). Male pigs remained fully intact and piglets were weaned at day 28 ± 1.0 of lactation.

To study the residual effect of the preweaning dietary treatment in progeny, a subsample of 566 pigs (8.1 ± 1.31 kg) were selected at weaning. Within sow treatment groups, pens of 10 to 12 pigs of the same sex (entire male or female) of even weight were formed and blocked by sow treatment, sex, and BW. Pen groups for CONTROL (*n* = 12), DPS (*n* = 12), LMR (*n* = 12), and LMR + S (*n* = 12) were moved to weaner accommodation at weaning. Pig BW, feed disappearance, and health were monitored up to target slaughter weight. Weaner pens were equipped with fully slatted plastic floors (2.5 × 2 m) with automatic environmental control. Each pen had a shelf-type single-space (33 cm) wet-dry feeder (BA19100, Verba, Verbakel, The Netherlands) with inset nipple drinker and a supplementary bowl drinker (SS Drinker, Rotecna, Lleida, Spain). A spiked rubber ball (Easyfix Luna 142, Easyfix, Galway, Ireland) was provided for each pen as environmental enrichment. Temperature in the weaner rooms was maintained at 28 °C during the first week after weaning and reduced by 2 °C each week to 22 °C at the end of 4 wk. Ventilation was from a punched ceiling with air exhausted via a variable speed fan linked to a thermostat which was controlled by computer (Big Dutchman 135). At day 47 postweaning, pen groups were moved to finisher accommodation. Finisher pens had fully slatted concrete floors (2.4 × 4.2 m) with automatic environmental control. Each pen had one shelf-type single-space (33 cm) wet-dry feeder (MA19100, Verba) with inset nipple drinker and a supplementary bowl drinker (SS Drinker, Rotecna). A wooden (larch) post was provided for each pen as environmental enrichment. All rooms were equipped with windows for natural light. Temperature in the finisher rooms was maintained at 22 to 20 °C with the same type of ventilation system used as in the weaner house. Pigs in each treatment group were slaughtered over 2 wk when they reached the target slaughter weight of approximately 120 kg live weight (average age at slaughter 157 d). The heaviest pigs in each pen group were slaughtered during the first week and the remaining pigs in the pen were slaughtered 7 d later.

### Diet Preparation and Feeding

Diets were formulated to meet or exceed National Research Council (NRC, 2012) recommendations. Diet samples were analyzed for dry matter (DM; oven drying), ash (furnace drying and gravimetry), crude protein (Dumas method), total fat (Weibul acid hydrolysis), and crude fiber (Ankom 200 fiber analyzer, Macedon, New York, United States) by Sciantec Analytical Services Ltd, Selby, United Kingdom according to European Union Commission Regulation No 152/2009 of 27 January 2009 (European Commission, 2009). The ingredient composition and nutrient content of the diets are shown in Table 1. During gestation, sows were fed a gestation diet (diet 1) in meal form at a feed allowance of 2.2 kg/d between days 0 to 90 of gestation. From day 90 of gestation to parturition, gestation feed allowance was increased to 2.7 kg/d. In the farrowing room, sows were fed a lactation diet (diet 2; Table 1) in meal form using a computerized feed delivery system (DryExact Pro, Big Dutchman). Sows were fed twice daily from farrowing to day 6 of lactation and three times daily from day 7 to weaning at 28 d. Sows were fed according to a lactation feeding curve which started at 60 MJ DE/d at day 0 of lactation and gradually increased to 107, 125, 133, and 137 MJ DE/d at days 7, 14, 21, and 26 of lactation, respectively. During lactation, feed allocation for individual sows was adjusted up and down from the curve, as necessary, to ensure that sow feed intake was as close as possible to ad libitum feed allowance and to prevent feed wastage. Between weaning and service, sows were provided with ad libitum access to a lactation diet for 4 d followed by a gestation diet (diet 1; Table 1) in meal form. Water was provided on an ad libitum basis to sows from a single-bite drinker in the feed trough and to suckling piglets from a bowl in the farrowing pen.

**Table 1.** Composition of the experimental diet (on an air-dry basis; kg/tonne)

Diet number diet type	1 Dry sow	2 Lactation	^1^3 Starter	4 Link	5 Weaner	6 Finisher
*Ingredients*						
Barley	759.7	259.7	50.0	68.4	495.9	410.5
Wheat	0	455.2	0	100.0	216.8	390.0
Maize	0	0	231.0	300.0	0	0
Soybean meal	76.2	179.8	143.4	186.9	163.2	165.0
Full fat soybean meal	0	0	130.8	70.0	50.0	0
Whey permeate	0	0	200.0	150.0	0	0
Skim milk powder	0	0	125.0	50.0	0	0
Soya hulls	125.3	0	0	0	0	0
Soya oil	14.0	66.0	85.0	38.2	40.0	11.0
Premix	1.5^2^	1.5^2^	3.0^3^	3.0^3^	3.0^3^	1.0^4^
l-Lysine HCl	2.3	5.0	6.2	6.7	5.9	4.3
dl-Methionine	0.4	1.5	3.6	3.2	2.2	1.0
l-Threonine	1.0	2.7	3.7	3.4	2.7	1.9
l-Tryptophan	0	0.8	1.4	1.3	0.6	0.2
l-Valine	0	2.7	1.3	1.3	0.6	0
Limestone flour	8.5	11.5	7.0	7.5	10.5	11.0
Mono dicalcium phosphate	7.0	8.5	5.5	7.0	5.5	1.0
Salt	4.0	5.0	3.0	3.0	3.0	3.0
Phytase^5^	0.1	0.1	0.1	0.1	0.1	0.1
*Chemical composition*						
Dry matter^6^	883.0	864.0	912.0	904.0	890.5	880.0
Crude protein^6^	117.0	157.0	182.0	174.0	162.5	163.0
Ash^6^	47.0	50.0	58.0	51.0	44.0	46.5
Ether extract^6^	34.8	86.4	120.9	83.4	74.8	36.6
Crude fibre^6^	83.0	30.0	18.0	19.5	31.0	33.5
Lysine^7^	7.8	11.5	16.2	15.0	13.0	10.9
Methionine^7^	2.4	3.9	7.0	6.1	4.7	3.4
Cystine^7^	2.5	3.0	2.7	2.9	3.1	3.1
Threonine^7^	5.6	8.3	10.9	10.1	8.8	7.6
Tryptophan^7^	3.66	3.36	2.66	2.22	1.54	2.79
Digestible energy (MJ/Kg)^7^	13.20	15.20	16.20	15.00	14.27	13.73
Net energy (MJ/Kg)^7^	8.59	10.93	12.06	10.94	10.30	9.80
SID lysine^7^	6.6	10.7	15.3	14.1	12.0	10.0
Total calcium^7^	7.2	8.3	8.2	7.5	7.4	6.5
Digestible phosphorus^7^	3.5	3.8	4.6	4.2	3.3	2.5

^1^The starter diet was used as solid creep feed. It was also used to prepare the liquid mixture supplemented to piglets preweaning.

^2^Premix provided per kilogram of complete diet (diets 1 and 2): Cu from copper sulfate, 15 mg; Fe from ferrous sulfate monohydrate, 70 mg; Mn from manganese oxide, 62 mg; Zn from zinc oxide, 80 mg, I from calcium iodate, 0.6 mg; Se from sodium selenite, 0.2 mg; vitamin A as retinyl acetate, 3.44 mg; vitamin D3 as cholecalciferol, 25 mg; vitamin E as dl-alpha-tocopheryl acetate, 100 mg; vitamin K, 2 mg; vitamin B12, 15 μg; riboflavin, 5 mg; nicotinic acid, 12 mg; pantothenic acid, 10 mg; choline chloride, 500 mg; Biotin, 200 μg; folic acid, 5 mg; vitamin B1, 2 mg; and vitamin B6, 3 mg.

^3^Premix provided per kilogram of complete diet (diets 3, 4, and 5): Cu from copper sulfate, 100 mg; Fe from ferrous sulfate monohydrate, 90 mg; Mn from manganese oxide, 47 mg; Zn from zinc oxide, 120 mg; I from potassium iodate, 0.6 mg; Se from sodium selenite, 0.3 mg; vitamin A as retinyl acetate, 2.1 mg; vitamin D3 as cholecalciferol, 25 μg; vitamin E as dl-alpha-tocopheryl acetate, 100 mg; vitamin K, 4 mg; vitamin B12, 15 μg; riboflavin, 2 mg; nicotinic acid, 12 mg; pantothenic acid, 10 mg; choline chloride, 250 mg; vitamin B1, 2 mg; and vitamin B6, 3 mg.

^4^Premix provided per kilogram of complete diet (diet 6): Cu from copper sulfate, 15 mg; Fe from ferrous sulfate monohydrate, 24 mg; Mn from manganese oxide, 31 mg; Zn from zinc oxide, 80 mg; I from potassium iodate, 0.3 mg; Se from sodium selenite, 0.2 mg; vitamin A as retinyl acetate, 0.7 mg; vitamin D3 as cholecalciferol, 12.5 µg; vitamin E as dl-alpha-tocopheryl acetate, 40 mg; vitamin K, 4 mg; vitamin B12, 15 µg; riboflavin, 2 mg; nicotinic acid, 12 mg; pantothenic acid, 10 mg; vitamin B1, 2 mg; vitamin B6, 3 mg.

^5^The diet contained 1,000 phytase units (FYT) per kg feed (RONOZYME HiPhos GT, DSM, Belfast, UK).

^6^Analyzed composition.

^7^Calculated composition.

SID lysine, standardized ileal digestible lysine.

Starter diet (diet 3; Table 1) when in dry form was fed as pellets (3 mm diameter pellets) as creep feed to suckling pigs from day 10 after birth until weaning using a circular creep feeder (Easy pan, Rotecna) placed at the bottom edge of the heat pad. Creep feed was provided frequently in small quantities to ensure freshness and to minimize wastage. Dry matter disappearance (DMd) was recorded at days 21 and 28 of lactation for DPS by weighing the total amount of dry starter diet provided to each pen during the period. The milk replacer powder (Opticare, Swinco, Helmond, The Netherlands) used in treatments LMR and LMR + S was probiotic-free. The milk replacer powder contained the following, in descending order of inclusion: sweet whey powder, vegetable oils, porcine dried plasma powder, whey powder, digestible starch, dextrose, hyper-immunized egg powder, soya protein concentrate, hydrolyzed wheat gluten, premix of amino acids, vitamins, and trace minerals. The milk replacer powder contained 11.9 MJ/kg net energy, 21.5% crude protein, 9% fat, 0.1% crude fiber, 6.5% crude ash, 1.8% lysine, 0.46% methionine, 0.7% calcium, 0.55% phosphorus, and 0.7% sodium. Liquid milk replacer was prepared at a mixing ratio of 150 g of milk replacer powder per 1 L of water and fed through an automatic feeder (Babyfeed, Schauer Agrotronic GmbH, Prambachkirchen, Austria). The liquid feeding trough was positioned to one side of the sow’s head at the front of the farrowing pen. For treatment LMR + S, liquid milk replacer was fed from days 3 to 6 of age followed by liquid milk replacer with an increasing proportion of starter diet (diet 3; Table 1) added to the mixture as weaning age approached (Supplementary Table S1). The liquid starter for the LMR + S treatment was prepared by mixing 200 g of dry pelleted starter diet with 1 L of warm water (55 °C) to achieve a feed-to-water ratio of 1:5. From days 3 to 6 of age, 100% liquid milk replacer was provided to suckling pigs. The percentage of liquid milk replacer in the mixture decreased to 80% from days 7 to 10, 60% from days 11 to 14, 40% from days 15 to 17, 20% from days 18 to 21, and 0% from days 22 to 28, while the percentage of liquid starter simultaneously increased.

Following weaning, pigs were fed a sequence of diets in accordance with their growth stage. Starter diet (diet 3; Table 1) was provided from weaning to day 6 postweaning, link diet (diet 4; Table 1) from days 6 to 17 postweaning, weaner diet (diet 5; Table 1) from days 17 to 47 postweaning, and a finisher diet (diet 6; Table 1) from day 47 postweaning to slaughter (approximately day 129 postweaning). All diets for postweaning pigs were provided dry as 3 mm diameter pellets and on an ad libitum basis. Pigs were inspected daily and any pigs demonstrating visual signs of illness were treated appropriately. Assessment of clinical signs of disease and treatment protocols were followed in accordance with farm protocol. All veterinary treatments were recorded including antibiotic and anti-inflammatory treatments.

### Preweaning Liquid Feeding System

The supplementary liquid milk replacer and the liquid mixture of milk replacer and starter diet were provided through an automatic feeder (Babyfeed). Fresh feed was prepared twice daily at 0835 and 1645 hours. Ingredients (milk replacer powder and starter diet) were mixed with water at 55 °C for approximately 10 minutes. Ten feeding cycles (each lasting approximately 2 h) were programmed between 0930 and 0400 hours. During each cycle the in situ trough sensors checked the amount of liquid feed present in the trough three times. Whenever the feed level was below the level of the sensor, the trough was detected as empty, and milk replacer or the liquid mixture of milk replacer and starter diet, depending on treatment, was delivered to the trough and the amount delivered to the trough was recorded in the system computer at each re-fill. Therefore, each pen could potentially have been supplied with milk replacer or the liquid mixture of milk replacer and starter diet, depending on treatment, up to 30 times in a 24 h period. Each day after the last feeding, the system was cleaned in closed circuit, which included mixing tanks and all of the pipelines, with a 1% acid solution (Deosan Acidbrite AG313, Diversey Europe Operations BV, Utrecht, The Netherlands). In addition, the system was cleaned once a week with a 0.5% solution of an alkaline detergent (AvalKsan Gold Standard CF, Carbon Group, Ringaskiddy, Ireland), to help remove lime scale from the circuit. The troughs were cleaned each morning with air pressure and rinsed with acidified water and a 0.5% solution of the alkaline detergent was applied to troughs, as for the acid rinse, once weekly. Every morning, before the cleaning of the troughs, any remaining uneaten feed was quantified in each trough. The pH of samples from the LMR tank and from the LMR + S tank was measured at 0900 and 1530 hours on days 4, 8, 12, 15, 18, and 25 of lactation for quality control purposes. The pH was reduced between morning and afternoon measurements (6.08 vs. 4.35 ± 0.166 pH units and 5.40 vs. 4.11 ± 0.179 pH units, for the LMR and LMR + S, respectively; Supplementary Table S2). The lactic acid bacteria count increased between morning and afternoon samples, especially for LMR + S (Supplementary Figure S1).

### Sow BW and BF Thickness

Sow BW and BF were recorded on day 110 of gestation, at weaning, and at their subsequent service (approximately day 4 postweaning). Sow BW was recorded using an electronic sow scales (EziWeigh 7i, O’Donovan Engineering, Co. Cork, Ireland). Empty farrowing weight was calculated using the following equation from the NRC (1998)^1^


SWfarr= [ SWd110(NB × 2.25)]1

where, SW_farr_, Empty sow farrowing weight, SW_d110_, Sow weight at day 110 of gestation, NB, total number of piglets born. The 2.25 kg is an estimate of the increased weight in the gravid uterus and in mammary tissue attributed to each pig in a litter (NRC, 1998).

Body fat was measured using a digital BF indicator (Renco LEANMEATER, Renco Corporation, Golden Valley, MN) by placing the probe of the digital indicator on the back of the sow at the level of the last rib, 6 cm to the side of the backbone. A reading was taken from the right and left side of the sow and the average reading was recorded.

### Farrowing Performance and Preweaning Piglet Growth Performance

The number of piglets born (total, live, stillborn, and mummified) was recorded for each litter at birth. The individual weight and sex of each piglet were recorded at birth, when each piglet was tagged for identification purposes, at 48 h after birth, on days 7, 11, 19, and 27 postpartum using an electronic piglet scale (Defender 3,000 XtremeW, O’Donovan Engineering). These data were used to determine the litter weight at each weighing, and piglet preweaning average daily gain (ADG). The coefficient of variation (CV) for within-litter BW was calculated for each weighing day. Piglet mortality between birth and weaning and between 48 h and weaning was also recorded. The average creep feed disappearance per litter was monitored on days 21 and 28 for DPS by weighing the total amount of dry starter diet provided to each pen. For LMR and LMR + S, the amount of liquid creep feed provided to each trough/pen was recorded daily by the system computer. The average creep feed disappearance per piglet was obtained by dividing the quantity provided per litter by the number of piglets alive in that litter at the recording time point.

### Live Observation of Trough-Directed Behavior Per Litter

The feeding behavior of individual piglets within pen groups was observed in batches 1 and 2 of the experiment using instantaneous scan sampling on days 12, 18, 22, and 26 after birth. To enable the easy identification of piglets during scan sampling, piglets in each litter were marked with a number from 1 to 17 (linked to their tag number) using black hair dye (Pro color plus, Healthpoint, Blackpool, United Kingdom) on the day before scan sampling was conducted. Six 1-hour sessions were conducted between 0900 and 1600 hours on each scan sampling day. During each 1-h session of live observations, each pen was scanned (i.e., the behavior of the group was recorded) every 3 minutes, leading to 21 scans/pen/session. A simple ethogram was used for scoring feeding behavior. At every scan, trough-directed activity (solid or liquid) was recorded. The trough-directed activity was defined as a piglet snout being inserted into the solid feed trough or immersed in the liquid feed for at least 2 s. Piglets were categorized as eaters if they were engaged in two or more trough-directed activities at any time during an observation day. The percentage of piglet eaters per pen was calculated on a pen basis for each observation day and for all observation days combined. This was carried out by dividing the number of piglets considered as eaters by the total number of piglets present in the pen, then multiplying the results by 100 to express as a percentage.

### Postweaning Pig Growth Performance

Pen groups were weighed on day 0 (weaning), 6, 14, 21, 28, 47 postweaning, and individual pig weights were recorded just prior to slaughter (at approximately day 129 postweaning) using an electronic scale (EziWeigh 7i, O’Donovan Engineering). Pigs were fasted prior to recording BW before slaughter. Feed disappearance was recorded on a pen basis between weaning and slaughter for the periods between which BWs were recorded. These data were used to determine the average daily feed intake, ADG, and Gain to Feed (G:F).

### Carcass Data

In total, 552 pigs (approximately 138/treatment) were transported 95 km to the abattoir (Dawn Pork & Bacon, Grannagh, Co. Waterford, Ireland) where they were killed by exsanguination after CO_2_ stunning. At the abattoir, carcass cold weight of individual pigs was calculated and muscle depth and BF were measured, as outlined in Arnaud et al. (2023). Lean meat content, carcass ADG, carcass G:F, and lean ADG were calculated as outlined in Arnaud et al. (2023).

### Intestinal Sampling and Small Intestinal Histology

A subset of 40 pigs (*n* = 10 per preweaning dietary treatment) were euthanized at day 5 postweaning by captive bolt followed by immediate exsanguination. After euthanasia, the entire intestinal tract was removed. Samples (approximately 2 cm) of tissue were excised from the duodenum (15 cm distal to the pyloric junction), jejunum (1.5 m distal to the pyloric junction) and ileum (15 cm proximal to the ileo-caecal junction). Tissue samples were rinsed in phosphate-buffered saline (Scientific Laboratory Supplies, Nottingham, United Kingdom) immediately post-harvest and placed in an alcohol/aldehyde fixative (No-Tox, Scientific Device Laboratory, Des Plaines, IL, USA) and that evening placed on a shaker for 48 h prior to storage at room temperature until histological analysis. Duodenal, jejunal, and ileal tissue samples were sent to an external company (NationWide Laboratories, Devon, United Kingdom). There, the tissue samples were removed from the No-Tox fixative and dehydrated through a graded alcohol series, cleared with xylene, and embedded in paraffin wax. Tissue samples were sliced into 5 µm sections using a microtome (Leica RM2135, Wetzlar, Germany), mounted on microscope slides and stained with hematoxylin and eosin for determination of gross morphological parameters of intestinal structure (villus height, villus width, and crypt depth and crypt width). For each pig, 10 villi and 10 crypts, where villi were attached to the lumen, were measured on five fields of view, and the means were used for statistical analysis.

### Health Monitoring

Fecal consistency scores on a pen basis were determined weekly on days 2, 7, 11, 19, and 27 before weaning and 6, 14, 21, and 28 postweaning. A 4-point scoring system (Casey et al., 2007) was used and the average score from five pigs was determined as the average score for each litter/pen. In brief: 0 = normal (dry pelleted feces), 1 = soft (soft with shape), 2 = mild diarrhea (very soft or viscous liquid), and 3 = severe watery diarrhea (watery or with blood). The diarrhea incidence at each time point was determined by considering a fecal score of 2 or greater as indicative of diarrhea for each litter/pen.

Antibiotic and anti-inflammatory usage were recorded in sows during lactation and in pigs from birth until they reached their target slaughter weight (separately for the weaner and finisher periods, respectively). Medication was administered when joint-ill, lameness, malaise, or diarrhea were observed in piglets and when malaise or vaginal discharge was observed in sows by trained farm technicians. One antibiotic (Unicillin, Univet Ltd, Cootehill, Co. Cavan, Ireland) and one anti-inflammatory (Loxicom, Norbrook, Newry, United Kingdom) only, were used during this experiment. Animal ID, pen number, product name, product code, dose administered (mL), frequency of administration, date of administration, and reason for use were recorded when an animal was treated. From this, the total number of piglet injections per litter/pen, the average volume of medication (antibiotic and anti-inflammatory) administered per pig on a litter basis and per sow, and the total number of clinical cases of disease (i.e., when an animal was treated one or more times) per litter were calculated preweaning. The average volume of medication (antibiotic and anti-inflammatory) administered per pig per pen was also calculated between weaning and target slaughter weight.

### Statistical Analysis

All data were tested for normality using the Univariate procedure and residuals were inspected in all models to confirm normality. Model fit was determined by choosing models with the minimum finite-sample-corrected Akaike Information Criteria.

All data were analyzed in Statistical Analysis Systems (SAS) using the linear mixed models procedure (PROC Mixed) using the software package version 9.4 (SAS Institute Inc., Cary, NC, United States), except the data for incidence of diarrhea. The incidence of diarrhea in the farrowing accommodation from days 2 to 28 and in the weaner accommodation from weaning to day 28 postweaning was analyzed using the PROC Glimmix procedure of SAS with a binomial distribution and the overall incidence reported for each of the pre- and postweaning periods. Data from batches 1, 2, 3, and 4 were analyzed together, as all measurements were recorded at the same time points.

For the analysis of preweaning litter weight, piglet BW, sow BW, and sow BF, the percentage of piglets classified as “eaters”, the total number of piglet injections per litter, the average volume of medication (antibiotic and anti-inflammatory) administered per pig on a litter basis and per sow, the total number of clinical cases of disease per litter, number of deaths and removals per litter, postweaning growth parameters, carcass quality data and the average volume of medication (antibiotic and anti-inflammatory) administered per pig on a pen basis postweaning and diarrhea incidence; treatment was included in the model as a fixed effect. For analysis of preweaning piglet growth parameters, piglet weight and litter size at 48 h were included as covariates, when significant in the model. For analysis of sow BW and BF, initial value at day 110 of gestation was included as a covariate in the model. For the analysis of postweaning growth and carcass quality parameters, weaning weight was included as a covariate. For BW at slaughter and cold carcass weight, the number of days from weaning to slaughter was included as a co-variate. Day was included in the above models as a repeated variable when relevant and block was included as a random effect. The litter/sow was the experimental unit for the analysis of all preweaning parameters, except piglet weight and growth, where the pig nested within sow/litter was the experimental unit. The experimental unit postweaning was the pen group.

For analysis of intestinal morphology parameters, treatment was included in the model as a fixed effect. Sow was included as a random effect and the pig was the experimental unit.

In all cases, differences in least square means were investigated using the *t*-test after Tukey adjustment for multiple comparisons. Results are presented in the text and tables as the least square means together with their pooled standard error. Differences between treatments were considered significant when *P* ≤ 0·05, whereas 0·05 < *P* ≤ 0·10 was considered as a tendency.

## Results

### Sow BW and BF Thickness

The effect of treatment on sow BW, BF depth, and reproductive performance from farrowing to service (approximately 4 d after weaning) is presented in Supplementary Table S3. There was no effect of treatment (*P* > 0.05) on any parameter of interest at any time point.

### Litter Size, Number Fostered, Pre- and Postweaning Deaths and Removals

The effect of treatment on sow litter size at birth, 48 h and weaning, the number of piglets fostered between 24 and 48 h and the number of piglet deaths per litter from birth to weaning and 48 h to weaning is presented in Supplementary Table S4. The total number of piglets born tended to be higher for treatment LMR compared to all other treatments (*P* = 0.06). The number of piglets born alive was higher for treatments LMR and LMR + S compared to CONTROL (*P* = 0.01). The number of piglets cross-fostered onto sows was higher in CONTROL compared to all other treatments (*P* = 0.05). There was no treatment effect (*P* > 0.05) on litter size at 48 h, litter size at weaning, number of deaths between birth and weaning and number of deaths between 48 h and weaning. Seven percent of all piglets on trial died between 48 h after birth and weaning. Among these, 52% died between days 2 and 7, 24% died between days 7 and 11, 14% died between days 11 and 19, and 10% died between days 19 and 28. Mortality rate from 48 h to weaning was 9%, 7%, 6%, and 6% for CONTROL, DPS, LMR, and LMR + S treatments, respectively. Deaths between days 2 and 6 were mainly due to starvation and crushing. After day 6, causes of mortality were more variable and included crushing, starvation, injury, and sudden death.

Two percent of all pigs on trial died or were removed postweaning. Among the dead or removed pigs, 50% died or were removed between weaning and day 47 postweaning and 50% died or were removed after day 47 postweaning. Postweaning mortality and removal rate was 6%, <1%, <1%, and 3% for CONTROL, DPS, LMR, and LMR + S treatments, respectively. Deaths and removals were due to lameness or injury.

### Preweaning Pig Growth Performance

The effect of treatment on piglet creep feed DMd, BW, and growth during the suckling period is presented in Table 2. Preweaning total DMd of creep feed per litter and per piglet was higher for DPS compared to LMR + S (*P* = 0.03). The DMd per litter and per piglet of the LMR treatment was similar to DPS and LMR + S (*P* > 0.05). There was no effect of treatment on litter weight at days 2, 7, 11, 19, and overall (*P* > 0.05). At day 27 of lactation, LMR piglets tended to have a higher litter weight than CONTROL piglets (*P* = 0.08). There was no effect of treatment on mean piglet BW at days 2, 7, 11, and overall (*P* > 0.05). At day 19 of lactation, LMR piglets tended to be heavier than DPS and LMR + S piglets (*P* = 0.06), whereas CONTROL piglets had a similar BW to all other treatments (*P* > 0.05). At day 27 of lactation, LMR piglets were heavier than piglets on all other treatments (*P* < 0.001), whereas DPS piglets were heavier than CONTROL piglets only. Piglets from the LMR + S treatment had a similar BW at weaning as CONTROL pigs and DPS pigs.

**Table 2.** Effect of dietary treatment on piglet weight and growth during the suckling period

Treatment	CONTROL	DPS	LMR	LMR + S	SEM	*P*-value
Number of sows	20	25	23	23		
*Litter weight, kg*						
Day 2	22.6	22.3	22.8	22.8	0.38	0.56
Day 7	36.6	35.0	36.0	37.3	1.13	0.48
Day 11	50.4	47.5	49.8	50.7	1.40	0.35
Day 19	79.8	76.0	81.9	81.7	2.09	0.15
Day 27	106.3^B^	108.0^A,B^	114.2^A^	111.8^A,B^	2.36	0.08
Overall					1.34	0.25
*Mean piglet BW, kg*						
Day 2	1.49	1.62	1.61	1.56	0.062	0.40
Day 7	2.54	2.59	2.57	2.58	0.063	0.95
Day 11	3.56	3.53	3.61	3.57	0.063	0.78
Day 19	5.81^A,B^	5.70^B^	5.91^A^	5.77^B^	0.063	0.06
Day 27	7.87^c^	8.10^b^	8.27^a^	7.99^b,c^	0.064	<0.001
Overall					0.056	0.27
*CV, %*						
Day 2	22	21	23	21	1.3	0.37
Day 7	21^b^	24^a,b^	25^a^	21^b^	1.3	0.03
Day 11	21^a,b^	24^a^	24^a^	20^b^	1.3	0.01
Day 19	22^a,b^	25^a^	24^a^	21^b^	1.3	0.02
Day 27	22^a,b^	25^a^	23^a^	20^b^	1.3	<0.01
Overall					0.90	<0.001
*ADG, g/pig/d*						
Days 2 to 7	187^b^	198^a^	193^a,b^	207^a^	4.8	<0.01
Days 7 to 11	243^a,b^	237^b^	263^a^	251^a^	5.5	<0.001
Days 11 to 19	251^b^	263^b^	280^a^	264^b^	5.4	<0.001
Days 19 to 27	223^c^	276^a^	275^a^	253^b^	5.8	<0.001
Overall					4.4	<0.001
*Dry matter disappearance* ^1^						
Preweaning creep feed, kg/litter	—	7.8^a^	6.6^a,b^	4.9^b^	0.74	0.03
Preweaning creep feed, g/pig	—	565^a^	471^a,b^	353^b^	51.4	0.03

^1^Dry matter disappearance is not applicable for CONTROL at any time point.

^a, b, c^Values within a row that do not share a common superscript are significantly different (*P* ≤ 0.05).

^A, B^Values within a row that do not share a common superscript tended to differ (0.05 < *P* ≤ 0.10).

CONTROL, control without supplementation; DPS, Dry pelleted starter diet; LMR, Liquid milk replacer; LMR + S, mixture of liquid milk replacer and liquid starter diet; BW, body weight; CV, coefficient of variation; ADG, average daily gain.

From days 2 to 7 of lactation, DPS and LMR + S piglets had a higher ADG than CONTROL piglets (*P* < 0.01), whereas LMR piglets had a similar ADG to all other treatments (*P* > 0.05). From days 7 to 11, LMR and LMR + S piglets had a higher ADG than DPS, but a similar ADG to CONTROL (*P* < 0.001). From days 11 to 19, LMR piglets had a higher ADG than for all other treatments (*P* < 0.001). From days 19 to 27, DPS and LMR piglets had a higher ADG than CONTROL piglets or LMR + S piglets (*P* < 0.001) and LMR + S piglets had a higher ADG than CONTROL piglets.

There was no treatment effect on the CV of within-litter BW at day 2 (*P* > 0.05). At day 7, CONTROL and LMR + S piglets had a lower CV than LMR piglets (*P* = 0.03). The LMR + S piglets had a lower CV than DPS and LMR piglets at day 11 (*P* = 0.01), 19 (*P* = 0.02), and 27 (*P* < 0.05); however, all treatments had a similar CV to that of CONTROL piglets.

### Live Observation of Trough-Directed Feeding Behavior Per Litter

The proportion of piglets having two or more feeder-directed activities within-litter is presented in Supplementary Table S5. There was no treatment effect on the percentage of eaters at day 12 of lactation. At days 18, 22, and 26 of lactation and overall, DPS had a higher percentage of eaters than all other treatments (*P* < 0.001). At day 22 and overall, LMR + S had a higher eater percentage than LMR only (*P* < 0.001).

### Preweaning Diarrhea Scores and Antibiotic and Anti-inflammatory Treatment

The effect of treatment on diarrhea incidence is presented in Table 3. There was no effect of treatment on diarrhea incidence between days 2 and 28 after birth (*P* > 0.05).

**Table 3.** Effect of dietary treatment on health parameters and medicinal treatment of sows and piglets during lactation

Treatment	CONTROL	DPS	LMR	LMR + S	SEM	*P*-value
Number of sows	20	25	23	23		
Diarrhea incidence in pigs, % (day 2-28)^1^	26.0	28.0	33.9	27.8	4.25	0.59
No. of clinical cases of disease per litter^2^	2.0	1.2	1.7	1.6	0.39	0.55
No. of injections per litter	6.6	3.8	4.5	4.7	1.30	0.50
Antibiotic usage per sow, mL^3^	16.9	9.3	4.2	4.9	4.51	0.20
Antibiotic usage per pig, mL^4^	0.2	0.1	0.2	0.2	0.04	0.46
Anti-inflammatory usage per sow, mL^5^	2.1	1.1	0.5	0.5	0.74	0.43
Anti-inflammatory usage per pig, mL^6^	0.04	0.02	0.02	0.03	0.009	0.65

^1^A fecal score of 2 or greater for a litter was considered indicative of diarrhea at each time point between days 2 and 28. The overall incidence was reported for the preweaning period.

^2^Number of piglets per litter treated one or more times up to weaning.

^3^Volume of antibiotic administered per sow between day 2 and weaning on day 28.

^4^Volume of antibiotic administered per piglet per litter between day 2 and weaning on day 28.

^5^Volume of anti-inflammatory administered per sow between day 2 and weaning on day 28.

^6^Volume of anti-inflammatory administered per piglet per litter between day 2 and weaning on day 28.

CONTROL, control without supplementation; DPS, Dry pelleted starter diet; LMR, Liquid milk replacer; LMR + S, mixture of liquid milk replacer and liquid starter diet.

The effect of treatment on the total number of clinical cases of disease per litter, the total number of injections per litter, and the average volume of medication (antibiotic and anti-inflammatory) administered per pig on a litter basis, and per sow, during the preweaning period, is presented in Table 3. There was no effect of treatment on any of these parameters (*P* > 0.05).

### Postweaning Pig Growth and Carcass Quality

The effect of treatment on pig growth, feed intake, and feed efficiency from weaning to slaughter is presented in Table 4. There was no effect of treatment on pig BW and average daily feed intake at any time point postweaning (*P* > 0.05). There was no effect of treatment on ADG from days 6 to 14, and on G:F ratio or ADG from days 14 to 21, 21 to 28, 28 to 47, 47 to slaughter, and overall. Compared to all other treatments, LMR pigs had a lower G:F ratio and ADG from weaning to day 6 postweaning (*P* < 0.001) and LMR + S piglets had a lower G:F ratio from days 6 to 14 postweaning (*P* < 0.001). The effect of treatment on carcass parameters is presented in Table 5. There was no effect of treatment on any parameter of interest (*P* > 0.05).

**Table 4.** Effect of dietary treatment on feed intake and growth of pigs from weaning to slaughter at approximately 120 kg

Treatment	CONTROL	DPS	LMR	LMR + S	SEM	*P*-value
Number of pens	12	12	12	12		
Days from weaning to slaughter	129	130	129	130	3.8	
*BW, kg*						
Day 0 (weaning)	8.1	8.1	8.0	8.1	0.31	0.99
Day 6 postweaning	9.2	9.2	8.9	9.2	0.35	0.98
Day 14 postweaning	12.2	12.2	11.8	11.9	0.43	0.96
Day 21 postweaning	15.5	15.6	15.0	15.5	0.60	0.93
Day 28 postweaning	18.8	19.2	18.5	19.3	0.82	0.73
Day 47 postweaning	32.1	31.9	31.3	32.7	1.72	0.48
Day of slaughter (approximately 157 d of age)	120.0	119.2	119.0	121.8	2.80	0.42
Overall					0.45	0.38
*ADFI, g/pig/d*						
Days 0 to 6	210	209	197	213	7.8	0.51
Days 6 to 14	382	410	384	412	17.5	0.48
Days 14 to 21	561	594	559	591	26.7	0.68
Days 21 to 28	715	735	679	741	36.1	0.62
Days 28 to 47	1,095	1,074	1,064	1,111	47.8	0.90
Day 47 to slaughter	2,149	2,225	2,232	2,226	92.5	0.91
Overall	847	871	850	882	31.8	0.87
*ADG, g/pig/d*						
Day 0 to 6	208^a^	198^a^	159^b^	191^a^	12.2	0.03
Day 6 to 14	370	371	357	343	18.7	0.65
Days 14 to 21	472	482	461	507	27.7	0.68
Days 21 to 28	482	526	489	546	24.2	0.19
Days 28 to 47	699	670	676	703	25.4	0.73
Day 47 to slaughter	1,095	1,076	1,087	1,092	17.1	0.84
Overall	554	552	537	663	11.8	0.42
*G:F, g/g*						
Days 0 to 6	1.00^a^	0.94^a^	0.83^b^	0.90^a^	0.035	<0.001
Days 6 to 14	0.98^a^	0.92^a^	0.94^a^	0.83^b^	0.033	<0.001
Days 14 to 21	0.83	0.81	0.83	0.86	0.033	0.60
Days 21 to 28	0.69	0.73	0.73	0.74	0.033	0.68
Days 28 to 47	0.64	0.63	0.64	0.63	0.033	0.99
Day 47 to slaughter	0.53	0.49	0.49	0.49	0.033	0.74
Overall	0.78	0.75	0.74	0.74	0.020	0.12

CONTROL, control without supplementation; DPS, Dry pelleted starter diet; LMR, Liquid milk replacer; LMR + S, mixture of liquid milk replacer and liquid starter diet; BW, body weight; ADFI, average daily feed intake; ADG, average daily gain; G:F, gain to feed ratio.

^a, b, c^Values within a row that do not share a common superscript are significantly different (*P* ≤ 0.05).

**Table 5.** Effect of dietary treatment on pig carcass parameters following slaughter

Treatment	CONTROL	DPS	LMR	LMR + S	SEM	*P*-value
Number of pens (pigs)	12 (128)	12 (143)	12 (141)	12 (140)		
Cold carcass weight, kg	91.7	91.2	91.7	93.1	0.99	0.51
Fat depth, mm	13.5	14.1	13.6	13.2	0.37	0.46
Muscle depth, mm	53.6	54.2	54.0	53.7	0.87	0.96
Lean meat, %	58.5	58.1	58.4	58.7	0.28	0.55
Kill out, %	76.2	76.7	76.8	76.3	0.35	0.59
Carcass ADG finisher, g/d^1^	870	861	868	876	13.4	0.87
Carcass G:F finisher, g/g^2^	0.44	0.40	0.40	0.40	0.017	0.15
Lean ADG, g/d^3^	653	646	652	664	12.5	0.78

^1^Carcass ADG (from day 47 postweaning to slaughter) = [(carcass weight in kg—day 47 weight in kg × 0.65) × 1,000]/number of days from 47 to slaughter (Lawlor and Lynch, 2005).

^2^Carcass G:F (from day 47 postweaning to slaughter) was calculated as follows: carcass G:F = carcass ADG (g)/daily feed intake (g).

^3^Lean ADG (from birth to slaughter) = (carcass weight × carcass lean meat percentage × 10)/number of days to slaughter (Lawlor and Lynch, 2005).

CONTROL, control without supplementation; DPS, Dry pelleted starter diet; LMR, Liquid milk replacer; LMR + S, mixture of liquid milk replacer and liquid starter diet; ADG, average daily gain; G:F, gain to feed.

### Postweaning Diarrhea Scores, Antibiotic and Anti-inflammatory Treatment

The effect of treatment on diarrhea incidence from weaning to day 28 postweaning is presented in Table 6. There was no effect of treatment on diarrhea incidence between weaning and day 28 postweaning (*P > *0.05). No scores higher than 0 were given after 28 d postweaning.

**Table 6.** Effect of dietary treatment on postweaning diarrhea incidence and medicinal treatment of pigs

Treatment	CONTROL	DPS	LMR	LMR + S	SEM	*P*-value
Number of pens	12	12	12	12		
Diarrhea incidence, % (weaning to 28 d postweaning)^1^	23	17	19	10	5.4	0.45
*Weaner period*						
Antibiotic usage per pig, mL^2^	0.09	0.02	0.04	0.06	0.039	0.62
Anti-inflammatory usage per pig, mL^3^	0.05	0.01	0.02	0.03	0.020	0.64
*Finisher period*						
Antibiotic usage per pig, mL^2^	0.17	0.11	0.33	0.33	0.167	0.71
Anti-inflammatory usage per pig, mL^3^	0.05	0.04	0.11	0.11	0.055	0.68

^1^A fecal score of 2 or greater for each pen was considered indicative of diarrhea at each time point from weaning to day 28 postweaning. The overall incidence was reported for the postweaning period.

^2^Volume of antibiotic administered to each pig on a pen basis.

^3^Volume of anti-inflammatory administered to each pig on a pen basis.

CONTROL, control without supplementation; DPS, Dry pelleted starter diet; LMR, Liquid milk replacer; LMR + S, mixture of liquid milk replacer and liquid starter diet.

The effect of treatment on the average volume of medication (antibiotic and anti-inflammatory) administered per pig on a pen basis postweaning is presented in Table 6. There was no treatment effect on the volume of antibiotic or anti-inflammatory administered per pig on a pen basis during the weaner or finisher periods.

### Intestinal Morphology

The effect of treatment on intestinal morphology at day 5 postweaning is presented in Table 7. There was no effect of treatment on villus height, crypt depth, villus height to crypt depth ratio (VH:CD), or villus width in the duodenum and jejunum of pigs. The DPS and LMR + S pigs tended to have greater villus height in the ileum than CONTROL piglets or those supplemented with LMR (*P* = 0.07).

**Table 7.** Effect of dietary treatment on small intestinal morphology of pigs at day 5 postweaning

Treatment	CONTROL	DPS	LMR	LMR + S	SEM	*P*-value
Number of pigs	10	10	10	10		
*Duodenum*						
Villus height, μm	332	363	393	363	21.8	0.29
Crypt depth, μm	218	226	254	225	20.7	0.61
VH:CD ratio, μm/μm	1.70	1.74	1.70	1.77	0.216	0.99
Villus width, μm	120	127	139	128	5.7	0.15
*Jejunum*						
Villus height, μm	286	337	319	300	16.8	0.18
Crypt depth, μm	203	239	256	221	19.9	0.28
VH:CD ratio, μm/μm	1.53	1.53	1.29	1.48	0.382	0.97
Villus width, μm	102	111	114	113	4.6	0.25
*Ileum*						
Villus height, μm	242^B^	284^A^	248^B^	283^A^	14.0	0.07
Crypt depth, μm	232	203	180	211	15.0	0.13
VH:CD ratio, μm/μm	1.09	1.47	1.43	1.44 44	0.368	0.19
Villus width, μm	111	119	115	121	3.8	0.31

CONTROL, control without supplementation; DPS, Dry pelleted starter diet; LMR, Liquid milk replacer; LMR + S, mixture of liquid milk replacer and liquid starter diet; VH:CD, villus height (μm)/ crypt depth (μm).

^A, B^Values within a row that do not share a common superscript tended to differ (0.05 < *P* ≤ 0.10).

## Discussion

This study is novel, in that it compares the effect of creep feeding dry pelleted starter diet, liquid milk replacer, and a liquid mixture of milk replacer and starter diet to suckling pigs on pre- and postweaning growth to target slaughter weight (approximately 120 kg) and medication usage. The results should provide valuable practical information for the design of optimal preweaning nutritional and management strategies to maximize pre- and postweaning pig growth.

### Preweaning

It was hypothesized that feeding creep feed in liquid form would increase the creep feed DMd of suckling piglets. However, this was not the case, as piglets supplemented with DPS had a higher total preweaning DMd (565 g/pig) than those supplemented with LMR + S (353 g/pig) while being similar to those supplemented with LMR (471 g/pig). In general, intake of dry pelleted creep feed in the current study was higher than that reported in previous studies (Bruininx et al., 2002; Byrgesen et al., 2021) and intakes of liquid creep were lower than that found in other studies (Lawlor et al., 2002; Wolter et al., 2002; Pustal et al., 2015). Based on recent work from our group (Vasa et al., 2023), it is likely that the frequency of sensor checks in the liquid feed troughs in the current study was insufficient, particularly during the final week prior to weaning, and this may have negatively impacted intake of liquid feed. Further to this, the feeder space of the liquid feeders was less than half that of the dry creep feeders (226 cm^2^ with a circumference of 34.5 cm vs. 573 cm^2^ with a circumference of 84.8 cm) and this may also have contributed to the lower liquid feed intake (Appleby et al., 1991). It is likely that increasing the proportion of milk replacer powder in the LMR + S mixture or feeding it for additional days during early supplementation would increase DMd and pig growth. However, milk replacer powder is approximately three times more expensive than starter diet and increasing its proportion in the mixture is likely to be uneconomic.

In the current study, preweaning provision of DPS and LMR increased piglet BW at weaning compared to the control, whereas this was not the case when LMR + S was provided. This is in agreement with previous findings where weaning weight was increased when DPS (Lee and Kim, 2018) or LMR (Wolter et al., 2002; Van Oostrum et al., 2016) were provided as creep feed. Despite LMR and LMR + S having similar preweaning DMd and LMR resulting in similar DMd to DPS, it was surprising that weaning weights were higher for LMR than for all other treatments and that litter weights tended to be higher for LMR than the control. The milk replacer powder in our study contained approximately 40% lactose and it could be that the higher intake of lactose contributed to the better preweaning growths observed when LMR was fed. Suckling piglets have high lactase activity in their small intestine and only a poorly developed capacity to digest starch and other substrates from plant-based ingredients (Pluske et al., 1997). Regarding the lack of response of LMR + S on weaning weight, others have also found that liquid creep feeding did not increase pig weight at weaning (Martins et al., 2020; Byrgesen et al., 2021; Vodolazska et al., 2023) even when DMd was higher than for dry creep feed (45 g/piglet vs. 25 g/piglet; Byrgesen et al. [2021]). In the current study, it might be that the period of supplementation with liquid milk replacer was too short (only 3 d) for the LMR + S treatment. Vodolazska et al. (2023) supplemented piglets for 10 d and did not observe an increase in weight at weaning. It is likely that liquid milk replacer should be supplemented for a longer period in order to increase weaning weight. In the current study, liquid creep feed treatments also had a higher number of live born than the control, suggesting that piglets were generally smaller in those litters. Although there was no significant growth improvement for piglets supplemented with the liquid mixture compared to the control, this suggests that results could have been poorer in these litters without the provision of the liquid mixture. In the current study, although not significant, medication usage in sows, clinical cases of disease, and number of injections per litter were numerically reduced in all of the creep feed treatments compared to the control which was not provided with creep feed. Additionally, deaths from 48 h to weaning for LMR and LMR + S were numerically reduced compared to the control (−0.4 pig deaths). These results, although not significant, suggest that creep feeding in dry or liquid form improves the health of suckling pigs, thereby reducing the need for medication and resulting in increased survival of pigs to weaning.

Others have shown that the growth response to creep feeding is largely influenced by the proportion of piglets within the litter that consume creep feed before weaning (i.e., “eaters”) as reviewed by Middelkoop (2020). It is interesting to note that the DPS diet not only resulted in higher preweaning DMd in the current study but the highest proportion of piglets within each litter categorized as “eaters” was also found for this treatment compared with the liquid feed treatments. Nonetheless, piglets supplemented with dry creep did not have the highest weaning weight which suggests that feed wastage may have been an issue with DPS. Every effort was made to minimize feed wastage in the current study. However, feeder design and accessibility can influence feed wastage (Tokach et al., 2020) and although the dry creep feeders used in the current study had plastic separators to stop the animals from lying in the feeder, they were open and had no hopper. This could have promoted creep wastage by allowing piglets to root in the feed, as suggested by Sulabo et al. (2010a). It was not expected that a lower percentage of piglets from the liquid creep feed treatments would be classified as “eaters” compared to those from the dry creep treatment. One could speculate that, as dry pelleted creep feed was always present in the feeders, piglets had frequent and smaller meals compared to those supplemented with liquid milk/mixture. This would have increased their chances of being observed in feeder-directed activity while live observations were being conducted. Further to this, as sensors on the liquid feed troughs were checked only three times every two hours, it is possible that troughs could have been void of feed for long periods of time during which live observations were being conducted. Both of these factors might explain the lower proportion of pigs classified as “eaters” where liquid as opposed to dry creep feed was provided in the current study.

### Postweaning

Supplementing suckling piglets with liquid milk replacer resulted in reduced growth and poorer feed efficiency during the first week postweaning despite these pigs having been heavier at weaning. A possible explanation for this might be that preweaning milk replacer provision to suckling piglets did little to expose them to the largely vegetable-based substrates that dominate postweaning diets. For this reason, the development of the enzyme secretory capacity necessary to utilize the vegetable-based components of postweaning diets, although not measured in the current study, may have been delayed in comparison to where a starter diet was provided as creep feed, as demonstrated by others (Hampson and Kidder, 1986; de Passillé et al., 1989). As milk replacer is rich in lactose, a higher lactase activity was likely present at weaning when it was provided as creep feed. In contrast, the development of the necessary enzyme secretory capacity for starch degradation, for example, was likely delayed in comparison to that for all other treatments, explaining the reduced growth observed with this treatment in the immediate postweaning period. It is likely that pigs familiarized with a liquid milk-based diet prior to weaning were less equipped to digest the plant-rich dry pelleted starter diet offered postweaning. This is supported by the reduced VH in the ileum of LMR pigs compared to those provided with DPS or LMR + S as a creep feed.

With a very high proportion of ‘eaters’ (78% of piglets observed having at least two feeder-directed activities at day 26 of age), very high total DMd per pig and increased weaning weight in pigs supplemented with DPS preweaning, a positive effect on intakes early postweaning and possibly postweaning growth may have been expected based on the work of Sulabo et al. (2010a). However, no increase in postweaning feed intake or growth was observed for pigs on this treatment compared to the control. On the other hand, the lack of postweaning response is not surprising considering that the proportion of ‘eaters’ was most likely over-estimated and feed wastage was increased on this treatment, as outlined above.

In the present study there was a tendency for increased VH in the ileum of piglets supplemented with DPS and LMR + S, at 5 days postweaning, compared to control and liquid milk replacer-fed pigs. The latter may indicate a positive effect of this supplementation on intestinal structure postweaning. The effects of creep feeding on postweaning intestinal morphology are not consistent in the literature, as they are dependent on feed intake and diet composition (Huting et al., 2021). As intake and growth of pigs supplemented with DPS and LMR + S were not increased early postweaning, the tendency for an increase in VH in the ileum in the current study must be interpreted with caution. This tendency to increase VH could be explained by the exposure to different sources of carbohydrates and plant-based proteins preweaning with these treatments. Soya was the main protein source of the starter diet, while whey and casein are the main protein sources of sow’s milk (Theil and Hurley, 2016). Despite the lack of a significant effect of preweaning creep feeding on medication usage and clinical cases postweaning, it is interesting to note that pigs supplemented with LMR + S had a numerically lower incidence of diarrhea (−13%) postweaning compared to the control. This is in agreement with Vodolazska et al. (2023) who observed an increase in fecal dry matter early postweaning when liquid feed had been provided to suckling pigs. Supplementing a liquid mixture of milk replacer and starter diet or dry starter diet alone could therefore have familiarized the pig to these indigenous ingredients preweaning, thereby limiting their damaging effect on the intestinal tract early postweaning. However, as outlined above, this did not result in a concomitant improvement in feed intake or growth.

The feed cost per pig during lactation based on the current study was €0.59, €0.71 and €1.41 per pig for DPS, LMR + S, and LMR, respectively. These costs do not include the capital and running costs of the liquid feeding system. Therefore, milk replacer powder is expensive and its use must be economically justified.

## Conclusion

The provision of liquid milk replacer to suckling piglets from days 3 to 28 after birth and dry pelleted starter diet from days 10 to 28 after birth increased piglet weaning weight. However, this weaning weight advantage did not persist postweaning and the slaughter weight of pigs on both treatments was similar to that of the control where no creep feed was provided. Providing a liquid mixture of milk replacer and starter diet did not result in increased growth pre- or postweaning compared to the control. Providing creep feed as a dry pelleted starter diet or as a liquid mixture of milk replacer and starter diet to suckling piglets tended to increase postweaning ileal VH. The management of the automated milk delivery system should be further optimized in order to ensure, in future studies, more frequent feed deliveries to troughs. This would increase liquid creep feed availability, and most likely increase the DMd of liquid creep-fed piglets. This, in turn, should result in greater benefits from liquid creep feeding of piglets.

## Supplementary Material

txae041_suppl_Supplementary_Materials
