# Supplementary material for: Effect of creep feeding pelleted starter diet, liquid milk replacer and a liquid mixture of starter diet and milk replacer to suckling pigs on their growth and medication usage
Source: Transl Anim Sci. 2024 Mar 16;8:txae041. doi: 10.1093/tas/txae041 (PMC11034430; doi:10.1093/tas/txae041)
Supplement: txae041_suppl_Supplementary_Materials [file txae041_suppl_supplementary_materials.docx]

# Supplemental material

**Table S1. Quantity of milk replacer powder, water and solid starter diet mixed and fed through the milk feeder system for the liquid mixture of milk replacer and liquid starter diet (LMR+S) experimental treatment.**

| **Experimental period** | **From 48 h to day 6** | **From day 7 to day 10** | **From day 11 to day 14** | **From day 15 to day 17** | **From day 18 to day 21** | **From day 22 to day 28** |
| --- | --- | --- | --- | --- | --- | --- |
| **Ingredients, g** |  |  |  |  |  |  |
| Milk replacer powder | 130.43 | 104.35 | 78.26 | 52.17 | 26.09 | 0 |
| Water | 869.57 | 862.32 | 855.07 | 847.82 | 840.58 | 833.33 |
| Starter diet | 0 | 33.33 | 66.67 | 100 | 133.34 | 166.67 |

**Table S2: pH evolution in the liquid milk replacer (LMR) tank and the liquid mixture of milk replacer and starter diet (LMR+S) tank immediately after feed preparation and just before the last feed of the day.**

| **Experimental day pre-weaning (piglets’ birth = day 0)** | **Day**  **4** | **Day**  **8** | **Day**  **12** | **Day**  **15** | **Day**  **18** | **Day**  **25** | **Mean** | **SD** |
| --- | --- | --- | --- | --- | --- | --- | --- | --- |
| **Liquid milk replacer (LMR) tank** |  |  |  |  |  |  |  |  |
| Morning pH, after feed preparation (0900 h) | 6.12 | 5.99 | 6.15 | 6.13 | 6.06 | 6.01 | 6.08 | 0.067 |
| Afternoon pH, before the last feed (1530 h) | 4.87 | 4.20 | 4.25 | 4.18 | 4.39 | 4.23 | 4.35 | 0.264 |
| **Liquid mixture of milk replacer and starter diet (LMR+S) tank** |  |  |  |  |  |  |  |  |
| Morning pH, after feed preparation (0900 h) | NA^1^ | 5.12 | 5.40 | 5.62 | 5.64 | 5.23 | 5.40 | 0.230 |
| Afternoon pH, before the last feed (1530 h) | NA | 3.95 | 4.00 | 4.15 | 4.22 | 4.23 | 4.11 | 0.128 |

^1^NA, not applicable, as at day 4 the liquid milk replacer provided in the LMR and LMR+S treatments was prepared from one mixing tank.

**Table S3. Effect of pre-weaning creep feed treatment on sow body weight and back-fat depth.**

| **Treatment^1^** | **CONTROL** | **DPS** | **LMR** | **LMR+S** | **SEM** | **P-value** |
| --- | --- | --- | --- | --- | --- | --- |
| Number of sows | 20 | 25 | 23 | 23 |  |  |
| Lactation length, days | 28.4 | 28.0 | 28.1 | 28.0 |  |  |
| **BW, kg^2^** |  |  |  |  |  |  |
| Day 110 of gestation | 274 | 274 | 274 | 274 | 2.4 | 0.99 |
| Farrowing^3^ | 239 | 238 | 232 | 235 | 2.4 | 0.11 |
| Weaning^4^ | 240 | 240 | 236 | 238 | 2.4 | 0.51 |
| Service^4^ | 229 | 230 | 226 | 228 | 2.6 | 0.68 |
| Overall |  |  |  |  | 1.8 | 0.38 |
| **BF, mm^5^** |  |  |  |  |  |  |
| Day 110 of gestation | 16.8 | 16.7 | 16.5 | 16.3 | 0.35 | 0.69 |
| Weaning | 13.0 | 13.0 | 13.4 | 13.5 | 0.35 | 0.58 |
| Service | 12.9 | 12.6 | 13.0 | 13.3 | 0.37 | 0.41 |
| Overall |  |  |  |  | 0.26 | 0.78 |
| **Sow BW change, kg** |  |  |  |  |  |  |
| Day 110 to weaning^6^ | -33.2 | -34.1 | -37.1 | -36.4 | 2.98 | 0.70 |
| Farrowing to weaning^7^ | 1.8 | 2.0 | 5.3 | 2.7 | 2.98 | 0.78 |
| Weaning to service^8^ | -7.7 | -7.6 | -7.1 | -8.4 | 3.29 | 0.99 |
| Overall |  |  |  |  | 2.14 | 0.96 |
| **Sow BF change, mm** |  |  |  |  |  |  |
| Day 110 to weaning^9^ | -3.7 | -3.8 | -3.1 | -2.9 | 0.37 | 0.22 |
| Weaning to service^10^ | 0.0 | -0.3 | -0.3 | -0.4 | 0.40 | 0.87 |
| Overall |  |  |  |  | 0.29 | 0.70 |

^1^CONTROL, control without supplementation; DPS, Dry pelleted starter diet; LMR, Liquid milk replacer; LMR+S, mixture of liquid milk replacer and liquid starter diet.

^2^BW, body weight.

^3^Estimated empty farrowing weight = (sow weight at day 110 – (total born × 2.25)). The value of 2.25 kg is an estimate of the increased weight in the gravid uterus and in mammary tissue attributed to each pig in a litter (NRC, 1998).

^4^Weaning = day 28 ± 1 of lactation; service = day 4 ± 1 post-weaning.

^5^BF, back fat.

^6^Sow BW change = (sow BW at weaning – sow BW at day 110 of gestation).

^7^Sow BW change = (sow BW at weaning – sow BW at farrowing).

^8^Sow BW change = (sow BW at service – sow BW at weaning).

^9^Sow BF change = (sow BF at weaning – sow BF at day 110 of gestation).

^10^Sow BF change = (sow BF at service – sow BF at weaning).

**Table S4. Effect of treatment on sow litter-size and the number of piglets fostered and died per litter during lactation.**

| **Treatment^1^** | **CONTROL** | **DPS** | **LMR** | **LMR+S** | **SEM** | **P-value** |
| --- | --- | --- | --- | --- | --- | --- |
| Number of sows | 20 | 25 | 23 | 23 |  |  |
| **Litter** |  |  |  |  |  |  |
| Total born^2^ | 15.6^B^ | 16.1^B^ | 18.8^A^ | 17.4^A,B^ | 0.92 | 0.06 |
| Live born | 13.8^b^ | 15.2^a,b^ | 17.8^a^ | 16.9^a^ | 0.90 | 0.01 |
| Litter size at 48 h | 14.5 | 14.5 | 14.9 | 14.7 | 0.20 | 0.40 |
| Litter size weaning | 13.3 | 13.4 | 13.9 | 14.0 | 0.31 | 0.29 |
| **Deaths and removals per sow** |  |  |  |  |  |  |
| Cross fostered from 24 h to 48 h^3^ | 2.2^A^ | 0.6^A,B^ | -0.9^B^ | -0.5^B^ | 0.83 | 0.05 |
| Deaths total | 2.7 | 2.5 | 2.9 | 2.4 | 0.45 | 0.86 |
| Deaths after 48 h | 1.2 | 1.2 | 0.8 | 0.8 | 0.23 | 0.36 |

^1^CONTROL, control without supplementation; DPS, Dry pelleted starter diet; LMR, Liquid milk replacer; LMR+S, mixture of liquid milk replacer and liquid starter diet.

^2^Total number born = number of piglets born alive, stillborn, and mummified.

^3^ Cross fostered from 24 h to 48 h = piglets cross fostered on – piglets cross fostered off; a minus indicates that more were fostered off than fostered on to the sow.

^A, B^ Values within a row that do not share a common superscript tended to differ (0.05 < P ≤ 0.10).

**Table S5:** **Effect of treatment on percentage of “eaters” within a litter where two or more feeder-directed activities per pig are considered to indicate an “eater”.**

| **Treatment^1^** | **CONTROL** | **DPS** | **LMR** | **LMR+S** | **SEM** | **P-value** |
| --- | --- | --- | --- | --- | --- | --- |
| **Eaters, %^2^** |  |  |  |  |  |  |
| Day 12 of lactation | - | 3 | 10 | 13 | 5.7 | 0.39 |
| Day 18 of lactation | - | 48^a^ | 18^b^ | 19^b^ | 5.7 | <0.001 |
| Day 22 of lactation | - | 76^a^ | 17^c^ | 35^b^ | 5.7 | <0.001 |
| Day 26 of lactation | - | 78^a^ | 24^b^ | 30^b^ | 5.7 | <0.001 |
| Overall | - | 51^a^ | 17^c^ | 24^b^ | 3.3 | <0.001 |

^1^CONTROL, control without supplementation; DPS, Dry pelleted starter diet; LMR, Liquid milk replacer; LMR+S, mixture of liquid milk replacer and liquid starter diet.

^2^The percentage of piglet eaters per pen was calculated on a pen basis for each observation day and for all observation days combined. This was carried out by dividing the number of piglets considered as eaters (having two or more feeder-directed activities) by the total number of piglets in the pen, then multiplying the results by 100 to express as a percentage. The percentage of observations of piglets seen engaging in trough-directed activity is not applicable for CONTROL at any time point.

^a, b, c^ Values within a row that do not share a common superscript are significantly different (P ≤ 0.05).

**Figures**


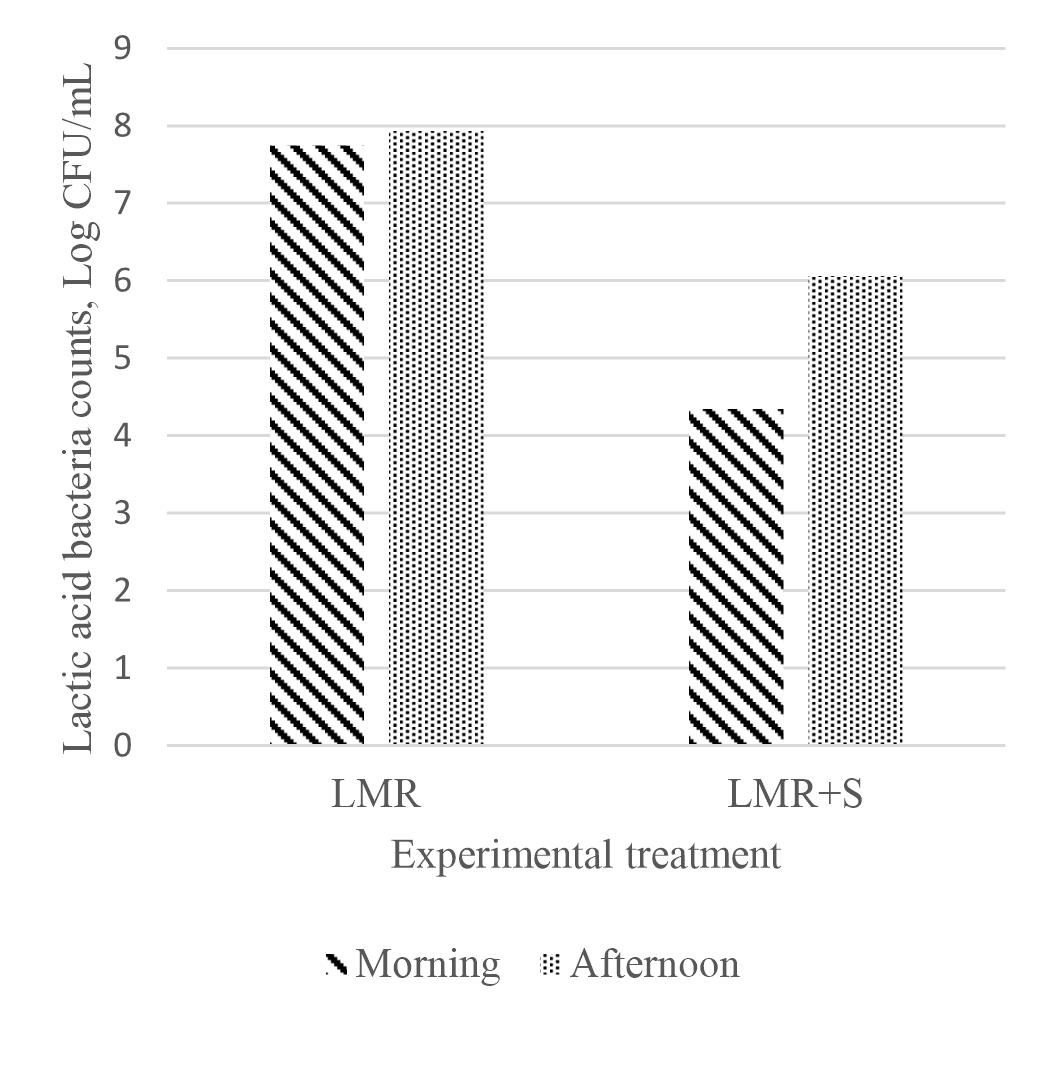


**Figure S1. Lactic acid bacteria counts (Log CFU/ml) in the liquid milk replacer (LMR) and the liquid mixture of milk replacer and starter diet (LMR+S) immediately after preparation in the morning (at 0900 h) and just before the last feed in the afternoon (at 1530 h). Counts were taken at day 15 pre-weaning.**
